# The Evaluation of Diabetic Patients' Awareness of Diabetic Retinopathy and Its Complications in the Western Region of Saudi Arabia

**DOI:** 10.7759/cureus.53090

**Published:** 2024-01-28

**Authors:** Abdulaziz Aldahlawi, Loujen Alamoudi, Nada Taher, Ahmed N Alnabihi, Naif Almufarriji, Reham Alzahrani, Karim Talat

**Affiliations:** 1 Medicine Department, King Saud Bin Abdulaziz University for Health Sciences College of Medicine, Jeddah, SAU; 2 Medical Research Department, King Abdullah International Medical Research Center, Jeddah, SAU; 3 Surgery/Ophthalmology Department, King Faisal Specialist Hospital and Research Centre, Riyadh, SAU; 4 Emergency Department, King Abdullah International Medical Research Center, Jeddah, SAU; 5 Emergency Department, King Saud Bin Abdulaziz University for Health Sciences College of Medicine, Jeddah, SAU; 6 Ophthalmology Department, King Saud Bin Abdulaziz University for Health Sciences College of Medicine, Jeddah, SAU; 7 Ophthalmology Department (Vitroretinal Sugery), King Abdulaziz Medical City, Jeddah, SAU

**Keywords:** health education and awareness, type 1 and type 2 diabetes mellitus, ophthalmology, diabetic eye disease, diabetic retinopathy

## Abstract

Background

Diabetes mellitus (DM) is a chronic metabolic disorder characterized by hyperglycemia. Globally, 382 million people have diabetes mellitus, and 90% of these patients suffer from type 2 diabetes. Saudi Arabia has the second-highest prevalence of diabetes among all Middle Eastern countries. Diabetic retinopathy (DR) is a significant complication of diabetes; early detection and proper intervention are important for its management and prognosis.

Aim

This study aims to assess the awareness of diabetic patients of diabetic retinopathy symptoms and complications in the western region of Saudi Arabia.

Methods

This is a cross-sectional study in which a convenience sampling technique was implemented for collecting data from all patients who fulfilled the inclusion criteria within the timeframe between February 2022 and October 2022 among diabetic patients at the National Guard Health Affairs in the western region. We included both type 1 and type 2 diabetic participants who are older than 18 years of age and have at least one visit to any outpatient ophthalmology clinic.

Results

This study involved 259 participants. The mean age of the participants is 46.69 (standard deviation {SD}: 15.59). Type 2 was more prevalent among the applicants (58.3%). A total of 242 (93%) participants were aware that diabetes could affect their eyes. Surprisingly, 130 (50.2%) do not know about diabetic retinopathy therapy options. The most significant obstacle to being examined early for ophthalmological diseases among diabetic patients was the deficient knowledge of diabetic retinopathy. Also, a significant statistical relationship was found between the year of diagnosis and the level of awareness regarding eye complications.

Conclusion

Despite the high level of awareness of diabetic retinopathy among diabetic patients shown in our study, it did not correspond to a high level of self-awareness on the importance of preventive measures such as annual diabetic retinopathy screening.

## Introduction

Diabetes mellitus (DM) is a chronic metabolic disorder characterized by hyperglycemia. It is defined as an elevation in glucose concentrations in the blood. This condition can be a result of either a defect in insulin secretion or a resistance against insulin hormone. Among the various types of diabetes mellitus, type 1 and type 2 are the most common [1]. In 2013, it was estimated that 382 million people had diabetes mellitus globally; approximately 90% of these patients suffer from type 2 diabetes. This number is equivalent to 8.3% of adults [2]. Saudi Arabia has the second-highest prevalence of diabetes among all Middle Eastern countries and ranks seventh globally [3]. Unfortunately, the prevalence is expected to further increase due to various different factors such as a sedentary lifestyle, aging, and the growing obesity rates in Saudi Arabia [4]. This endocrinological disease has diverse complications in multiple organ systems, significantly contributing to its high morbidity and mortality rates, one of which is microvascular diabetic retinopathy (DR) [5], which is attributed to about 4.8% of blindness causes globally [6].

Diabetic retinopathy is a significant complication of diabetes. It is deemed a leading cause of preventable blindness in working-age adults [6]. According to the Global Burden of Disease Study, DR is the fifth leading cause of blindness in adults aged 50 years or older [7]. Globally, the prevalence of DR among diabetic patients is estimated to be around 34% [8]. In Saudi Arabia, the prevalence of DR is very common, as reported in multiple articles from different regions around the country. For instance, in Al-Madinah city, the prevalence of diabetic retinopathy is estimated to be 36%. Similarly, in the Al-Ahsa region, a prevalence of 33% is reported. In the southern region, 27.8% of diabetic patients developed DR [9-11]. The major risk factors that accelerate the development and progression of DR include high blood glucose, long-standing DM, and high blood pressure. Therefore, adequate lifelong control of glucose levels delays and reduces the progression of DR [12].

Early detection and proper intervention could be enhanced through sufficient awareness and appropriate knowledge regarding such a highly prevalent disease. A study has shown a significant correlation between insufficient risk factor control and the patients' lack of awareness in regard to their disease. Conversely, positive attitudes and good practice patterns were observed in educated patients with above-average knowledge about their condition [13]. This study aims to assess the awareness of diabetic patients of diabetic retinopathy in the western region of Saudi Arabia.

## Materials and methods

This time-limited cross-sectional study, implanting a convenience sampling technique for data collection, was conducted on the patients affiliated with the National Guard Health Affairs in the western region of Saudi Arabia from February 2022 to October 2022. To ensure the novelty of our data, data collection was confined within the specified timeframe. The study focused on assessing all diabetic patients, encompassing both type 1 and type 2 individuals, aged 18 and above, who had attended at least one outpatient ophthalmology clinic visit during the study period.

Ethical considerations were paramount in the study design. Approval was diligently obtained from the Institutional Review Board (IRB) of the King Abdullah International Medical Research Center (approval number: IRB/1464/23), ensuring adherence to established ethical guidelines and the protection of participants' rights and well-being. All participants provided informed consent before inclusion in the study, emphasizing the importance of respecting autonomy and ensuring voluntary participation.

A validated questionnaire, adapted from a previously published article, was utilized for data collection [14]. This questionnaire, along with the informed consent form, was transformed into a Google Form (Google, Inc., Mountain View, CA) and distributed to the patients via their mobile numbers to capture their responses. Initially, the questionnaire covered demographic data, diabetes characteristics, glycemic control, and cardiovascular medical history. It subsequently delved into the participants' awareness regarding diabetic retinopathy. The collected data were electronically aggregated into a spreadsheet for subsequent analysis.

The questionnaire was organized into four distinct sections. The first section aimed to capture the participants' demographic information, combining qualitative and quantitative data, including gender, marital status, nationality, education level, and age. The second section delved into the specifics of the participants' diabetes, seeking information on the type of diabetes they had and the number of years since their initial diagnosis. The third section probed the participants' past medical history, covering significant aspects such as a history of hypercholesterolemia, hypertension, and ischemic heart disease, providing insights into the participants' overall health background. The fourth section consisted of patient-reported questions designed to gauge the participants' perceptions and knowledge regarding diabetes and eye health. These questions addressed the participants' evaluation of blood glucose control, awareness of the ocular impact of diabetes, opinions on the necessity of regular eye examinations for controlled and uncontrolled diabetes, identification of complications related to uncontrolled hyperglycemia, and knowledge of available therapy methods for diabetic retinopathy. Additionally, the participants were queried on how they became aware of diabetes-related complications, the reasons prompting their initial eye examination, and perceived obstacles hindering early examination for ophthalmological diseases.

For statistical analysis, the spreadsheet was imported into JMP statistical software version 15.2.0 (SAS Institute Inc., Cary, NC) (a subsidiary of the SAS Institute). Qualitative variables were expressed as frequencies and percentages, while mean and standard deviation (SD) were used to represent quantitative outcomes. Inferential statistics synthesis was carried out exclusively using the chi-square test, with a significance level set at a p-value lower than 0.05.

## Results

Baseline

A total of 259 participants responded to this study's questionnaire in the western region of Saudi Arabia (Jeddah, Makkah, and Madinah), most of whom were females (60.6%). The mean age of our participants is 46.69 (SD: 15.59). Of the respondents, 45.2% graduated from college. Type 2 was more prevalent among the applicants (58.3%), with the highest duration of year since diagnosis being 6-10 years (27%). Regarding blood glucose control, medium control was more prominent among our patients, notably among females (102). Out of the 259 participants, 147 (56.8%) have a history of hypercholesterolemia, 114 (44%) have a history of hypertension, and 25 (9.65%) have a history of ischemic heart disease (Table 1).

**Table 1:** Baseline of participation SD: standard deviation

Parameter	Categories	Total, N (%)
Age	Mean (±SD)	46.69 (±15.59)
Gender	Male	102 (39.4%)
	Female	259 (60.6%)
Diabetes type	Type 1	108 (41.7%)
	Type 2	151 (58.3%)
Year since diagnosis	More than 20 years	63 (24.3%)
	11-20 years	58 (22.4%)
	6-10 years	70 (27%)
	Less than five years	68 (26.3%)
History of hypercholesterolemia	Yes	147 (56.8%)
	No	112 (43.2%)
History of hypertension	Yes	114 (44%)
	No	145 (56%)
History of ischemic heart disease	Yes	25 (9.65%)
	No	234 (90.3%)
Nationality	Saudi	231 (89.2%)
	Non-Saudi	28 (10.8%)
City	Jeddah	165 (63.7%)
	Makkah	50 (19.3%)
	Madinah	33 (12.7%)
	Bahrah	11 (4.25%)
Educational level	College	117 (45.2%)
	Postgraduate	12 (4.63%)
	High school	69 (26.6%)
	Secondary	24 (9.27%)
	Primary	15 (5.79%)
	No education	22 (8.49%)

Patient awareness of diabetes-related complications and how to deal with them

Out of the 259 participants, 242 (93%) are aware that diabetes could affect their eyes, and 107 (41.3%) think that even people with controlled diabetes could be affected by diabetes-related eye disease. Regarding the awareness of other diabetes-related complications, 127 (49%) chose coronary artery disease, 107 (41.3%) chose cerebrovascular disease, 102 (39.4%) chose peripheral vascular disease, 187 (72.2%) chose neuropathy, and 197 (76.1%) chose nephropathy. The most common source of information selected by the participants was physicians, nurses, and ophthalmologists. Moreover, 173 (66.8%) participants consider that people with good control of diabetes should have their eyes examined on a regular basis, while 228 (88%) see that people with uncontrolled diabetes should have their eyes examined on a regular basis. In addition, 136 (52.5%) were compelled to have their eyes examined initially by their physician. Surprisingly, 130 (50.2%) do not know about diabetic retinopathy therapy options. The most significant obstacle to being examined early for ophthalmological diseases among diabetic patients was deficient knowledge of diabetic retinopathy.

Participant responses based on their year of diagnosis

The year of diagnosis and awareness about eye complications were statistically significant (P=0.0348); six (8.82%) participants of those diagnosed less than five years were not aware that diabetes could cause eye disease, while those who were diagnosed with diabetes for more than 20 years were aware. Moreover, 43 (63.24%) of those who were diagnosed with diabetes for less than five years are not aware of the available therapy methods for diabetic retinopathy, while only 22 (34.92%) of those who were diagnosed for more than 20 years are unaware (Table 2). 

**Table 2:** Participants' responses based on their year of diagnosis

Awareness assessment	Responses	Less than five years, N (%)	6-10 years, N (%)	11-20 years, N (%)	More than 20 years, N (%)	P-value
How do the subject evaluate his/her blood glucose control?	Excellent	22 (32.35%)	19 (27.14%)	11 (18.97%)	13 (20.63%)	0.4102
	Medium	40 (58.82%)	38 (54.29%)	37 (63.79%)	40 (63.49%)	
	Weak	6 (8.82%)	13 (18.57%)	10 (17.24%)	10 (15.87%)	
Is the subject aware that diabetes could affect his/her eyes?	Yes	58 (85.29%)	65 (92.86%)	56 (96.55%)	63 (100.00%)	0.0348
	No	6 (8.82%)	3 (4.29%)	2 (3.45%)	0 (0.00%)	
	I do not know	4 (5.88%)	2 (2.86%)	0 (0.00%)	0 (0.00%)	
Is the subject aware of the available therapy methods for diabetic retinopathy?	Glycemic control	17 (25%)	22 (31.43%)	14 (24.14%)	15 (23.81%)	0.0038
	Laser therapy	3 (4.41%)	12 (17.14%)	12 (17.14%)	19 (30.16%)	
	Surgery	5 (7.35%)	2 (2.86%)	2 (2.86%)	7 (11.11%)	
	He/she does not know	43 (63.24%)	34 (48.57%)	34 (48.57%)	22 (34.92%)	

## Discussion

The current study measured the awareness of diabetic patients about diabetic retinopathy. In the present study, the mean age of the patients was 46.69 years old, with 60.6% being females, and the majority (58.3%) had type 2 DM. This study revealed that almost 93% were aware that diabetes has an effect on the eyes, which is similar to the findings of another cross-sectional study conducted among diabetic Saudi patients between 15 and 75 years of age (80.8%) [14]. However, it was significantly lower than the data from a study conducted in India, where only 37.1% of the participants, who were rural Indian residents, confirmed their awareness of the effect of DM on the eyes [15]. Such discrepancy in awareness can be explained by the participants' level of education and the quality of the provided health services. This is supported by the fact that the respondents with higher education levels, similar to the respondents in the current study where 45.2% had graduated from college, tended to have better DR awareness compared to the participants with lower education levels, such as the rural population [16].

In addition to the timely diagnosis of DR by early screening programs, the tight control of glycated hemoglobin (A1C) is one of the crucial modifiable risk factors for controlling DR progression among DM patients [12]. In the present study, 66.8% of the participants believe that individuals with controlled DM should have regular eye checkups. Moreover, 88% see that people with uncontrolled diabetes should have their eyes examined on a regular basis. This, in fact, highlights that the participants of the present study have an adequate level of knowledge about DR, which could be attributed to the long duration of their DM that may have resulted in cumulative knowledge gained over years and many encounters or hospital visits/follow-ups. A recent study conducted among diabetic Saudi participants showed that 59% of the participants with well-controlled blood glucose believed in the necessity of DR screening and follow-up, even with controlled A1C. On the other hand, only 29.7% of the poorly controlled group think that there is a need to screen the eye with good control (P<0.0001) [14]. This, indeed, sheds light on the importance of education with special and extra emphasis on patients with poorly controlled comorbidities.

The annual routine eye examination is the recommended practice for diabetic patients as a preventive measure for diabetic retinopathy. Despite the high level of awareness of diabetic retinopathy, in the present study, approximately half (52.5%) of our study subjects were examined initially due to a physician referral, whereas only 34.7% of the patients sought examination based on their knowledge of diabetic retinopathy. This finding was similarly reported by Alzahrani et al. [17]. However, a higher level (70.9% and 93.3%) of self-awareness in diabetic retinopathy screening was reported in two studies conducted in India [15,18]. This emphasizes the role of physicians and other healthcare providers in our community to consider regular referrals to an ophthalmologist for diabetic screening and providing education about the importance of regular eye examinations.

Among the complications of diabetes, eye diseases were reported the most, which is consistent with the reported findings in a previous study [17]. The main source of this knowledge was reported to be by healthcare workers (physicians, nurses, and ophthalmologists), in contrast to a study from Bangladesh that showed that the two most important sources were friends and neighbors (51%) and family (49%) [19]. Regarding the patients' knowledge about available treatments for diabetic retinopathy, most of our patients (50.2%) were unaware of the treatment options, which is consistent with another study conducted in Riyadh [20]. This lack of knowledge was most commonly observed among diabetic patients who had been diagnosed within five years (63.24%) (P<0.003), possibly due to limited exposure to healthcare education by physicians. A small portion of the diabetic patients believed that diabetic retinopathy could be treated with laser (15.8%) and surgery (7.72%). Inadequate knowledge was identified as the primary reason for not undergoing eye examinations, which aligns with a previous study by Srinivasan et al. [13]. Future education interventions in our society should focus on raising awareness of the importance and frequency of screening.

This study had some limitations regarding its type. The questionnaire was constructed as closed-ended, which lacks an in-depth view of patient knowledge and perception. In addition, distributing the questionnaires via WhatsApp (Meta Platforms, Inc., Menlo Park, CA) limits the reliability of the information given by the subjects. The main strength of our study is that the patients included were from the whole western region instead of only patients attending eye clinics or primary healthcare centers as in previous studies, which yields a better representation of the awareness and eye practice of diabetic patients in society. Furthermore, our study examined other factors that could affect the patients' knowledge about diabetic retinopathy such as educational level and disease duration.

## Conclusions

Despite the high level of awareness of diabetic retinopathy among diabetic patients shown in our study, it did not correspond to a high level of self-awareness on the importance of preventive measures such as annual diabetic retinopathy screening. Educational level and the disease duration were important factors for the level of knowledge on diabetic retinopathy. Multicentered studies are needed to investigate different patient populations to have tailored educational programs targeting multiple age groups. Moreover, more studies are needed to assess the challenges diabetic patients face; thus, this in turn will lead to efficient and high-quality counseling sessions to handle all patients' concerns.
